# Rhizobactin B is the preferred siderophore by a novel *Pseudomonas* isolate to obtain iron from dissolved organic matter in peatlands

**DOI:** 10.1007/s10534-020-00258-w

**Published:** 2020-10-07

**Authors:** Stefan Kügler, Rebecca E. Cooper, Johanna Boessneck, Kirsten Küsel, Thomas Wichard

**Affiliations:** 1 Institute for Inorganic and Analytical Chemistry (IAAC), Friedrich Schiller University Jena, 07743 Jena, Germany; 2 Institute of Biodiversity, Friedrich Schiller University Jena, 07743 Jena, Germany; 3 The German Centre for Integrative Biodiversity Research (iDiv), Halle-Jena-Leipzig, 04103 Leipzig, Germany

**Keywords:** Dissolved organic matter, Iron, Metallophore, *Pseudomonas*, Peatland, Siderophore

## Abstract

Bacteria often release diverse iron-chelating compounds called siderophores to scavenge iron from the environment for many essential biological processes. In peatlands, where the biogeochemical cycle of iron and dissolved organic matter (DOM) are coupled, bacterial iron acquisition can be challenging even at high total iron concentrations. We found that the bacterium *Pseudomonas* sp. FEN, isolated from an Fe-rich peatland in the Northern Bavarian Fichtelgebirge (Germany), released an unprecedented siderophore for its genus. High-resolution mass spectrometry (HR-MS) using metal isotope-coded profiling (MICP), MS/MS experiments, and nuclear magnetic resonance spectroscopy (NMR) identified the amino polycarboxylic acid rhizobactin and a novel derivative at even higher amounts, which was named rhizobactin B. Interestingly, pyoverdine-like siderophores, typical for this genus, were not detected. With peat water extract (PWE), studies revealed that rhizobactin B could acquire Fe complexed by DOM, potentially through a TonB-dependent transporter, implying a higher Fe binding constant of rhizobactin B than DOM. The further uptake of Fe-rhizobactin B by *Pseudomonas* sp. FEN suggested its role as a siderophore. Rhizobactin B can complex several other metals, including Al, Cu, Mo, and Zn. The study demonstrates that the utilization of rhizobactin B can increase the Fe availability for *Pseudomonas* sp. FEN through ligand exchange with Fe-DOM, which has implications for the biogeochemical cycling of Fe in this peatland.

**Electronic supplementary material:**

The online version of this article (10.1007/s10534-020-00258-w) contains supplementary material, which is available to authorized users.

## Introduction

Siderophores are small weight molecules produced endogenously by microorganisms, fungi, or plants under iron (Fe)-limited conditions or taken up from the surrounding environment (Neilands 1981). Once siderophores are secreted into the environment, they contribute to the organism´s ligandosphere, which describes the entirety of excreted metal complexing agents and ligands derived from the DOM (Deicke et al. 2019). Currently, more than 500 siderophores produced by various plants, fungi, or microorganisms under Fe-limited conditions have been identified (Hider and Kong 2010), which can also be facilitated for the recruitment of various essential trace elements (Kraepiel et al. 2009). Siderophores can be categorized according to their moieties as catecholates, hydroxamates, carboxylates, and diazeniumdiolates (Ahmed and Holmstrom 2014; Hermenau et al. 2018). Early pioneering studies identified Fe-carriers with combined functional groups (Leong and Neilands 1982). For example, pyoverdine, produced by many members of the *Pseudomonas* genus, contains three parts, a dihydroxyquinoline core, an amino acid peptide chain that varies among strains, and a small dicarboxylic acid (or their monoamides). These substances often harbor a mix of hydroxamate and catecholate functional groups (Meyer 2000).

*Pseudomonas* spp. belong to a genus of gram-negative bacteria, which are ubiquitous in soils and aquatic ecosystems (Moore et al. 2006; Palleroni 1993). In these habitats, Fe and organic carbon are closely associated through the complexation with dissolved organic matter (DOM) or the formation of Fe-OM aggregates (Riedel et al. 2013). The Fe-DOM complexes and Fe-OM aggregates can thus control the accessibility of Fe for the microorganism, especially in carbon-rich habitats such as peatlands, despite the high Fe-concentrations (Kügler et al. 2019; Thomas Arrigo et al. 2016). Peatlands store approximately 30% of the land-based organic matter and thus more than any other vegetation type in the world (Bragazza et al. 2013; Mitra et al. 2005). DOM can stabilize both ferrous (Fe^II^) and ferric (Fe^III^) Fe independent of the oxygen content in these environments (Bhattacharyya et al. 2018; Kügler et al. 2019). The co-occurrence of both Fe-redox states thus implies an essential role of microbial-mediated Fe-cycling in soils and peatlands. Both microbial Fe^II^-oxidation and Fe^III^-reduction are enhanced in the presence of Fe-DOM complexes by either inhibiting abiotic Fe^II^-oxidation or enhancing Fe-accessibility mediating electron transfer processes (Cooper et al. 2017; Hädrich et al. 2019; Kügler et al. 2019). Similarly, microbial Fe-assimilation mediated by siderophores might also be affected, as up to 97% of the Fe occurs in complexed form in peatlands (Kügler et al. 2019).

Our study thus aims to identify bacterial siderophores released by *Pseudomonas* spp. As a model organism, we used a *Pseudomonas* strain isolated from the Schlöppnerbrunnen fen, a well-studied peatland fed by Fe^II^-rich groundwater in the Northern Bavarian Fichtelgebirge (Germany). *Pseudomonas* spp. are known to produce several other siderophores, including enantio-pyochelin (Youard et al. 2007), quinolobactin (Mossialos et al. 2000), ornicorrugatin (Matthijs et al. 2008) or pre-pseudomonine (Özkaya et al. 2015). Most of the *Pseudomonas* strains, such as *P.* *fluorescens*, produce one of these secondary siderophores in addition to the primary siderophore pyoverdine (Cornelis 2010). Pyoverdines are released under strong Fe-limitation, as they are very effective in Fe-recruitment (Dumas et al. 2013; Ross-Gillespie et al. 2015). Secondary siderophores can play interesting and unique roles in the biological or chemical activities of *Pseudomonas* spp., such as inflammation, biodegradation processes, as well as plant defense processes and antibiosis (Cornelis 2010; McRose et al. 2018). In this context, siderophores are also known to complex other metals such as Cu, Mo, W, or Zn for trace metal uptake (metallophores) or detoxification (Kraepiel et al. 2009; Johnstone and Nolan 2015; McRose et al. 2018; Wichard et al. 2008).

Over the past decades, several advances have been achieved to identify siderophores. Whereas improved spectrophotometric (Perez-Miranda et al. 2007) and microbial bioassays (Soria-Dengg et al. 2001) provide a tool for fast, mostly qualitative, siderophores-surveys, high-performance liquid chromatography/electrospray ionization mass spectrometry (HPLC–ESI–MS) based protocols were established for the quantification of known siderophores (Kilz et al. 1999; McCormack et al. 2003). The development of high-resolution mass spectrometry (HR-MS) using the natural Fe-isotopic pattern became a powerful tool to identify candidates of siderophores (Baars et al. 2016; Lehner et al. 2013). In this study, metal isotope-coded profiling (MICP) was applied to screen for unique isotopic signatures of the equally added amounts of Fe-isotopes, ^54^Fe, and ^58^Fe, in HR-MS (Deicke et al. 2014; Wichard 2016).

As the Fe-species of the fen soil are controlled by DOM (Kügler et al. 2019), the development of strategies for acquiring Fe from the peat water is essential for the peat microbial community. Thus, we hypothesized that our isolate, *Pseudomonas* sp. FEN, releases siderophores to manage the Fe-acquisition from the DOM. Using MICP, we screened the cell-free supernatant collected from *Pseudomonas* sp. FEN cultures grown under Fe-limited conditions for metallophores, and the structure of the isolated candidate siderophore was elucidated. Ligand exchange and short-term uptake experiments with *Pseudomonas* sp. FEN were performed to demonstrate the potential physiological function of the identified novel siderophore.

## Materials and methods

### Chemicals

Water, methanol, and acetonitrile for extraction and LC were UHPLC grade and purchased from VWR Chemicals (Darmstadt, Germany). MilliQ water (18.2 MΩ cm) was obtained from a MerckMillipore purification system (Darmstadt, Germany). Fe, Cu, Zn, and Mo-isotopes were purchased from Eurisotop (Saint-Aubin, France). Pyoverdine (from *P. fluorescens* strain ATCC 13,525) was obtained from EMC Microcollections (Tübingen, Germany). All other chemicals for growth media were purchased from Alfa Aesar (Ward Hill, MA, United States), AppliChem (Darmstadt, Germany), Roth (Karlsruhe, Germany) and Sigma Aldrich (Taufkirchen, Germany).

### Sample material and cultivation

*Pseudomonas* sp. FEN was isolated from the Schlöppnerbrunnen fen in the Fichtelgebirge (Northern Bavaria, Germany) (Litzba 2009). *Pseudomonas* sp. FEN cultures were grown in PS medium overnight (ATCC medium 3: Nutrient broth medium) with shaking at room temperature. For incubation experiments, an aliquot (1 mL overnight 100 mL^−1^ media) of *Pseudomonas* sp. FEN overnight culture was transferred to serum bottles with modified Wolfe’s mineral medium (MWMM; ATCC medium 2672) containing 1 g NH_4_Cl, 0.2 g MgSO_4_·7H_2_O, 0.1 g CaCl_2_·2H_2_O, 0.05 g K_2_HPO_4_, amended with 10 mmol L^−1^ sodium lactate and 1 mL L^−1^ vitamin B solution (ATCC medium 2672). The pH of the media was adjusted to 6.5 with 10 mmol L^−1^ sodium hydrogen carbonate flushed with carbon dioxide before inoculation.

### Growth curve of *Pseudomonas* sp. FEN

Triplicate *Pseudomonas* sp. FEN cultures were incubated in trace metal free MWMM (V = 100 mL) in serum bottles at 24 ± 1 °C for 42 h in a shaking incubator. To monitor the growth of *Pseudomonas* sp. FEN*,* optical density (OD_600_) was measured in polystyrene cuvettes (Sarstedt, Nümbrecht, Germany) using a UV–Vis spectrometer (Thermo Fischer Scientific, Waltham, USA). Each sample was measured in triplicate.

### Phylogenetic analysis

Average nucleotide identity (ANI) values were calculated using the ANI-Matrix genome-based distance matrix calculator (Rodriguez-R and Konstantinidis 2016). The genome sequences for all *Pseudomonas* spp. were retrieved from the NCBI Assembly database in the form of nucleotide FASTA files, except for the newly sequenced *Pseudomonas* sp. FEN genome. *Pseudomonas* spp. strains used for the ANI comparisons were chosen based on the following two criteria: type of siderophore produced and the availability of the genome sequence in public databases. The ANI-matrix output was subsequently used for hierarchical clustering of the input genomes. The resulting tree is unrooted and derived using the BIONJ clustering method, a variant of the neighbor joining clustering method. The ANI-distance clustering tree, in Newick format, was visualized in MEGA X software (Stecher et al. 2020). Note, *Pseudomonas cepacia* ATCC25416 was renamed *Burkholderia cepacia* ATCC25416, and was used in the ANI-based analysis.

### Genomic DNA extraction, genome sequencing and analysis

*Pseudomonas* sp. FEN cultures were grown in PS medium overnight with shaking at room temperature. Genomic DNA extraction, genome sequencing, and analysis were performed as described in Cooper et al. (2020). Briefly, biomass was harvested via centrifugation (10 min, 5000 × *g*, 4 °C) and genomic DNA was extracted using the GenElute Bacterial Genomic DNA kit (Sigma Aldrich, Taufkirchen) according to manufacturer’s instructions. *Pseudomonas* sp. FEN genomic DNA was used for whole-genome sequencing (PacBio sequencing), based on the standard manufacturer’s protocol (Cooper et al. 2020). The newly sequenced genome was annotated using RASTtk with default parameters (Aziz et al. 2008; Brettin et al. 2015; Overbeek et al. 2014). For analysis of the genome sequence, tonB- dependent receptor homologs were detected using BlastP (protein–protein BLAST) and the non-redundant protein sequences (nr) database.

### Siderophore detection and identification using Chrome Azurol S and metal isotope-coded profiling

First, the chrome azurol S (CAS) assay, a universal colorimetric method, was applied in agar plates to detect siderophores independent of their structure. Siderophores scavenge Fe from the Fe-CAS-hexadecyltrimethylammonium bromide complex, and the free CAS dye changes from blue to yellow (Schwyn and Neilands 1987b). Agar plates prepared with PS medium were used to detect siderophore production by *Pseudomonas* sp. FEN and for the negative (abiotic) control.

To identify metallophores in the bacterial growth medium, cultures (V = 100 mL, OD_600_ = 0.2) were centrifuged (3500×*g* for 10 min at room temperature), and the supernatant was loaded on an HLB cartridge (225 mg, Oasis™, Waters, Milford, UK). Cartridges were conditioned with 3 mL methanol and equilibrated with 3 mL water, for solid-phase extraction (SPE). Compounds were eluted with 3 mL methanol (Deicke et al. 2014; Wichard 2016). The eluate was evaporated to dryness under nitrogen-steam and redissolved with 100 µL aqueous ammonium acetate (10 mmol L^−1^) at pH 6.6.

The identification of metal-binding complexes followed the workflow of metal isotope-coded profiling (MICP) identifying all peaks which show mass signals in a 1:1 intensity ratio and a mass difference of one of the applied isotope pairs (^54^Fe/^58^Fe = 3.9937u, ^63^Cu/^65^Cu = 1.9982u, ^66^Zn/^68^Zn = 1.9988u and ^95^Mo/^98^Mo = 2.9996u) (Deicke et al. 2014). Data was processed via DeltaMS, according to Baumeister et al. (2018). For example, to screen for siderophores, the Fe-isotopes ^54^Fe and ^58^Fe were solved in hydrochloric acid and were diluted with MilliQ water to 10^–2^ mol L^−1^. An isotope mixture containing equal amounts of ^54^Fe- and ^58^Fe-isotopes was prepared and added to the redissolved extract (10^–4^ mol L^−1^). The “isotope signature” type was chosen to identify isotopologues containing the isotopes ^54^Fe and ^58^Fe. The allowed variation of the isotope ratio was set to 10%. The following parameters were used for peak detection and DeltaMS processing: FWHM 30, steps 2, max 5, sntresh 5, step 0.01, mzdiffMatched 0.02, RTwindow 10, noiseCutoff 10, enriTol 0.1, varEQ false, numAtom 2, maxLT 4, ppmw 5, monoTol false, intChoice into, alpha 0.05, dpeak 12, compareOnlyDistros false.

### Mass spectrometry and nuclear resonance spectroscopy

Mass spectrometry were performed using the qExactive Plus Orbitrap (Thermo Scientific, Bremen, Germany) coupled with an Ultimate 3000 (Dionex, Sunnyvale, CA, USA) UHPLC. A Zorbax SB-C8 HPLC-column (150 × 4.6 mm; 1.7 µm; Agilent Technologies, Santa Clara, CA, USA) was used. Acetonitrile spiked with 10% water and 1 mmol L^−1^ ammonium acetate (B) and water spiked with 2% acetonitrile and 1 mmol L^−1^ ammonium acetate (A) were used as eluents. The gradient was started at 100% A for 0.2 min, ramped to 50% A at 4 min. 100% B was achieved at 4.2 min and was held for one minute followed by a throwback to 100% A until 6 min. This was held for 30 s.

Electrospray ionization was conducted in both negative and positive mode (ESI-/ESI+). The spray voltage was set to 3,300 V in negative ionization mode and 3,000 V in positive ionization mode, respectively. Capillary temperature was amounted to 360 °C, sheath gas flow to 60, aux gas flow to 20, sweep gas flow to 5 and AGC-target to 3 × 10^6^. Full scan analysis was conducted from *m*/*z* 100 to 1500 with a resolution of 70,000. The collision energy for MS/MS experiments, which were conducted in ESI + mode, was set to 30 eV at a resolution of 17,500 at *m*/*z* 200. The isolation window was set to 0.4 *m*/*z* for both compounds. All ion fragmentation of pyoverdine was executed in ESI+ mode with a resolution of 70,000. The collision energy was set to 75 eV, and the scan range from 50 to 750 *m*/*z*.

For nuclear magnetic resonance (NMR) analysis, purification of the siderophore candidates from 5 L culture medium using SPE was achieved by preparative HPLC as described in the next chapter, but without the addition of ^58^Fe. NMR-spectroscopy (^1^H,^13^C-HMBC and ^1^H,^13^C-HSQC) was performed with a 600 MHz Bruker Avance III using the residual resonance of the solvent D_2_O as an internal standard for reference.

### Preparation of the ^58^Fe-labelled siderophores

For short-term uptake experiments of siderophore candidates and subsequent analysis by inductively coupled plasma mass spectrometry (ICP-MS), a ^58^Fe-complex was prepared. ^58^Fe (3 mmol L^−1^) was added to the solid phase extract of the *Pseudomonas* sp. FEN supernatant. This procedure prevented ^56^Fe from being bound by the siderophore candidate, which was essential for the uptake experiments. To remove the excess of ^58^Fe, the ^58^Fe-complex was purified using an Agilent 1100 series HPLC system (Agilent Technologies, Santa Clara, CA, USA) and a non-end-capped Nucleosil® C8 HPLC column (250 × 21 mm, 5 µm, Macherey–Nagel, Düren, Germany). Water spiked with 2% acetonitrile (A) and 100% acetonitrile (B) were used as eluents. The gradient was started at 100% A for 1 min and was changed to 80% until 30 min elapsed. The fraction from 15 to 16 min was collected, and the solvent was removed using the vacuum centrifuge RVC 2–25 CP plus (Christ, Osterode am Harz, Germany). UHPLC-HR-MS monitored purification success.

### ^58^Fe-uptake experiments

Triplicates of *Pseudomonas* sp. FEN cultures (V = 25 mL, OD_600_ = 0.2) were centrifuged, washed three times with MWMM, and the cell pellets were resuspended in 25 mL of fresh medium. ^58^FeCl_3_, ^58^Fe-EDTA, ^58^Fe-pyoverdine, and ^58^Fe-rhizobactin B were added to the medium (pH was adjusted to pH 6.5), incubated for 2 min, and passed through a 0.45 µm cellulose nitrate filter (Sartorius, Göttingen, Germany).

The bacteria on the filter were rinsed immediately with each 25 mL 0.1 mol L^−1^ EDTA / 0.05 mol L^−1^ oxalate solution and 25 mL water. Filters were digested with 2 mL nitric acid (70%) at 70 °C for 1 h. 500 µL of the tuning solution (Agilent Technologies, Santa Clara, CA, USA) containing 10 µg L^−1^ yttrium was added to 150 µL of the sample, then diluted to 5 mL with water and were measured via ICP-MS. The measured ^58^Fe-quota was corrected by the ^58^Fe-concentration measured on the filter treated with the corresponding Fe-species only (i.e., abiotic controls). This value was normalized to the background concentration of ^58^Fe in cultures not amended with an exogenous ^58^Fe source.

### Inductively coupled plasma mass spectrometry

ICP-MS measurements were conducted to determine the concentration of ^58^Fe. The measurements were performed with the Agilent 7500c ICP-MS system (Agilent Technologies, Santa Clara, USA), equipped with a Babington nebulizer, a Scott spray chamber (cooled to 2 °C), and a Fassel torch. An ASX-500 autosampler (CETAC Technologies Inc., Ohama, USA) was connected to the ICP-MS with 1.02 cm ID PVC tubing for sample injection. The following settings were used for the ICP-MS measurements: RF power was set to 1,250 W, plasma gas flow set to 15 L min^−1^, and nebulizer gas flow set to 1.02 L min^−1^. The sample uptake time was 30 s (uptake speed: 350 µL min^−1^) and the rinse time 30 s.

### Peat water extraction and metal determination

Peat cores were taken from the upper 30 cm of the Schlöppnerbrunnen fen, transported to the laboratory, and processed within 3 h for peat water extractions. Briefly, 130 g (wet weight) peat was added to 1 L autoclaved MilliQ water, and the slurry was shaken at 400 rpm for 24 h at 4 °C. After the 24 h incubation period, the peat water extract (PWE) was filtered through 0.3 μm glass fiber filters (Whatman, Maidstone, Great Britain) and either used immediately or stored at 4 °C until further use. For ligand exchange experiments with the purified siderophore, the ligand was added to the PWE and incubated for 10 min at 20 ± 1 °C and at the pH of the PWE (pH 5.5). The UHPLC-HR-MS measurements were immediately performed after the ligand exchange to compare the peak areas. The metal content of the PWE was determined using atomic absorption spectroscopy (AAS) for Fe and ICP-MS for Al, Cu, Mo and Zn, according to Kügler et al. (2019).

### Monitoring of siderophore production during bacterial growth

For the growth-dependent production of rhizobactin B, 2 mL of the above-mentioned *Pseudomonas* sp. FEN cultures were sampled at the same time points as for the determination of the growth curve. SPE of the supernatant was performed using HLB cartridges (30 mg, Oasis™, Waters, Milford, UK), which were preconditioned with 1 mL methanol and equilibrated with 1 mL water before sample loading. Following the elution of rhizobactin B with 1 mL water, evaporating the water under nitrogen-stream, and dissolving the residue in water (40 µL), the purified rhizobactin B samples were analyzed by UHPLC-HR-MS.

### Statistical analysis

Statistical tests, including paired t-test and Dunnet’s test, were performed with Microsoft Excel 2016 (Redmont, WA, USA) and Minitab 16 (Minitab Inc., State College, PA, USA), respectively.

## Results

### Siderophore production by *Pseudomonas* sp. FEN

**Fig. 1:**
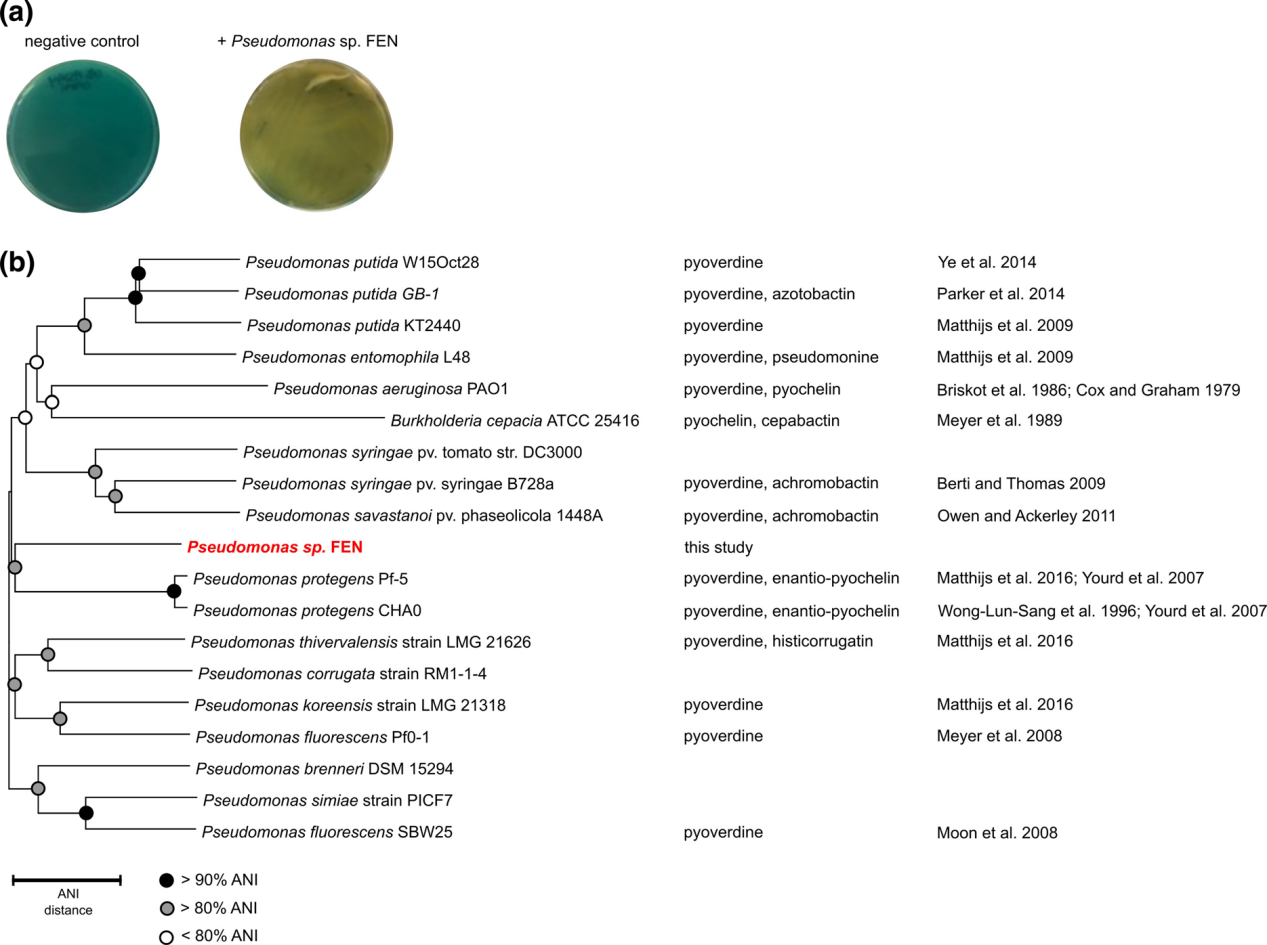
Characterization of the siderophore producing members of the family *Pseudomonadaceae*. **a** Siderophore production of *Pseudomonas* sp. FEN grown on PS plates with CAS. Images of PS-CAS plates incubated for 24 h prior to photography indicate *Pseudomonas* sp. FEN produces siderophores, compared to the negative (abiotic) control, as indicated by the color change in PS-CAS plates inoculated with *Pseudomonas* sp. FEN. The CAS assay begins blue and changes to yellow as the ligand exchange reaction between Fe^III^-CAS and competing siderophores occurs. The experiment was performed in triplicate. **b** ANI-distance clustering tree, in Newick format, based on pairwise ANI comparisons between various siderophore-producing *Pseudomonas* strains, including *Pseudomonas* sp. FEN isolated from the Schlöppnerbrunnen fen. ANI values are indicated by circles placed on the respective node. Black circles designate ANI values > 90%, grey circles designate ANI values > 80%, and open circles designate ANI values < 80%. The scale bar refers to the ANI distance. Identified siderophores and the corresponding references are given if MS and/or NMR measurements were available

In order to evaluate the production and secretion of siderophores, *Pseudomonas* sp. FEN was grown in PS media to exponential phase and transferred to PS-CAS plates for direct detection and visualization of siderophores. The CAS assay revealed that *Pseudomonas* sp. FEN can produce siderophores, as indicated by a color change from blue to yellow and thus indicating a ligand exchange reaction between Fe^III^-CAS and competing siderophores (Fig. 1a).

### Taxonomic classification of *Pseudomonas* sp. FEN

To date, *P. fluorescens* strains include a larger group of pyoverdine producers, some non-producers, and those with various moieties binding metals. Initial 16S rRNA-based analysis indicated the *Pseudomonas* sp. FEN isolate was most closely related to *P. fluorescens* strains. However, recent advances in genomics and taxonomic classifications, namely the implementation of average nucleotide identity (ANI)-based comparisons and standardized taxonomy classifications (Genome Taxonomy Database taxonomy) (Lalucat et al. 2020), revealed our isolate is more closely related to the genome of type strain *P. batumici*, rather than any of the publicly available *P. fluorescens* genome sequences that comprise the *P. fluorescens* phylogenetic group within the *P. fluorescens* lineage of the *Pseudomonadaceae* genera (data not shown). This taxonomic classification of our isolate, *Pseudomonas* sp. FEN, further explains why this strain does not produce any fluorescent siderophores, such as pyoverdine, but instead can only utilize them. The *Pseudomonas* strains selected for comparison produce pyoverdines as well as secondary siderophores such as achromonobactin, azobactin, cepabactin, histicorrugatin, pyochelin, pseudomonine and rhizobactin-like siderophore and have publicly available genomes. Using these publicly available genomes along with the newly sequenced *Pseudomonas* sp. FEN genome, we were able to calculate the average nucleotide identities between all genomes selected. The ANI-based comparisons showed the *Pseudomonas* sp. FEN isolate is most similar to *P. protegens* Pf-5 and *P. protegens* CHA0. Additionally, the ANI-distance clustering tree revealed that assemblage of these diverse *Pseudomonas* strains appears to have branched evolutionarily to form two closely related subgroups (clusters) (Fig. 1b). *Pseudomonas* sp. FEN fits into an evolutionary cluster with *P. savastanoi pv. phaseolicola* 1448A, *P. syringae* pv. syringae B728a*, P. syringae pv. tomato* str. DC3000, *B. cepacia* ATCC 25,416, *P. aeruginosa* PAO1, *P. entomophila* L48, *P. putida* KT2440, *P. putida* GB-1, *P. putida* W15Oct28, *P. protegens* Pf-5 and *P. protegens* CHA0, while *P. thivervalensis* strain LMG 21,626, *P. corrugata* strain RM1-1–4, *P. koreensis* strain LMG 21,318, *P. fluorescens* Pf0-1, *P. brenneri* DSM 15,294, *P. simiae* strain PICF7, and *P. fluorescens* SBW25 fit into another cluster (Fig. 1b). Based on the ANI calculations, *B. cepacia* ATCC 25,416 has the lowest overall ANI percentages for all pairwise comparisons and the pairwise comparison between *P. protegens* Pf-5 and *P. protegens* CHA0 showed the highest ANI percentages (ANI 99%).

### Siderophore screening

**Fig. 2:**
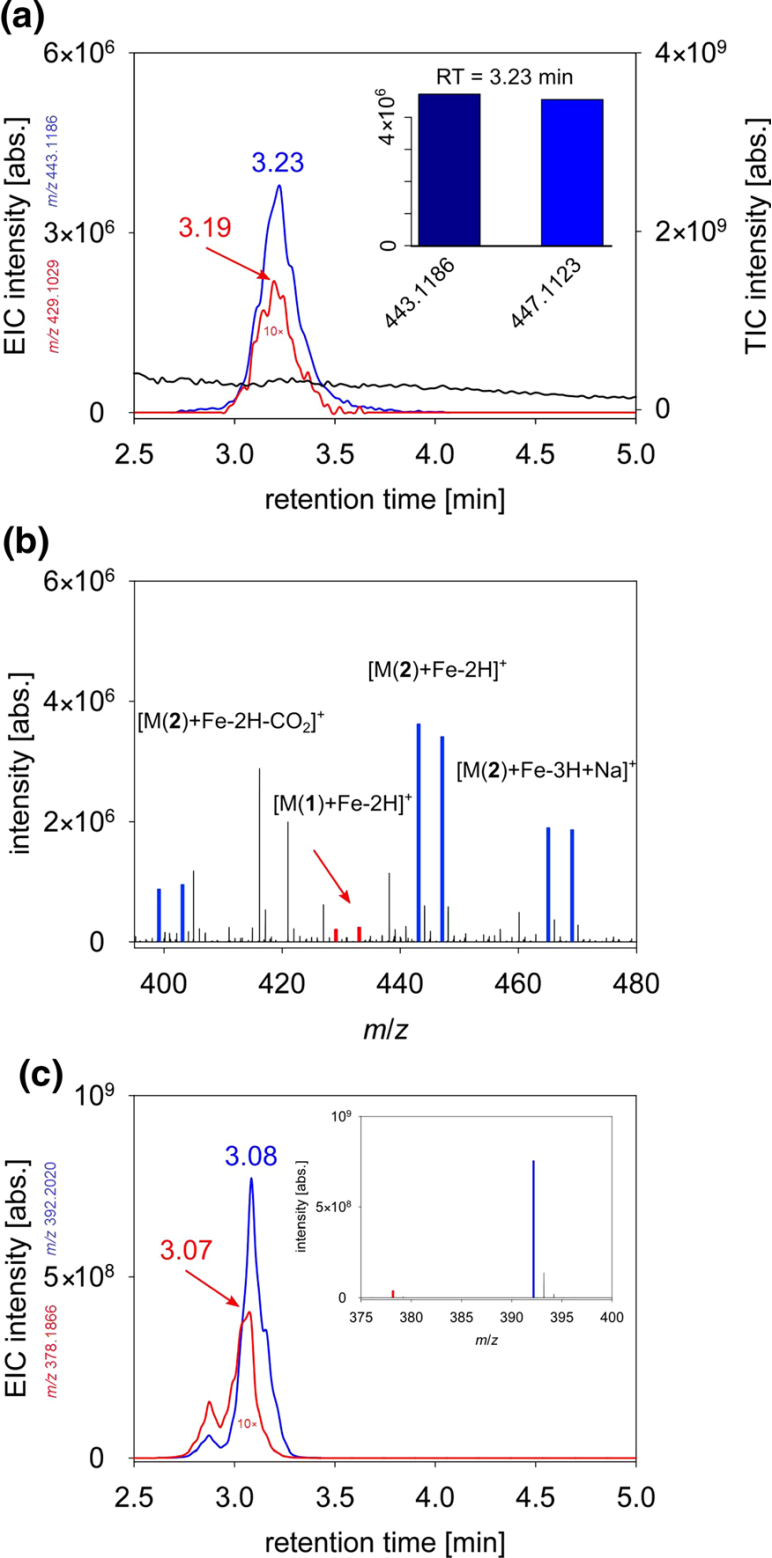
Identification of Fe-chelating compounds in *Pseudomonas* sp. FEN extract. **a** The total ion current (TIC, black line) and extracted ion chromatogram (EIC, blue and red lines) revealed two similar compounds, Fe-rhizobactin (**1**) (red line, intensity multiplied by factor 10) and elevated amounts of Fe-rhizobactin B (**2**) (blue line) detected by DeltaMS upon addition of stable pairs of the isotopes ^54^Fe and ^58^Fe in the positive electrospray ionization mode. Insert shows the readout of DeltaMS analysis at retention time 3.23 min: *m/z* 443.1186 and 447.1123. **b** The mass spectra reveals the characteristic isotopic signature of the quasi-molecule ions [M + H]^+^ of (**1**) *m/z* 429.1029; *m/z* 433.0965 and (**2**) *m/z* 443.1186; *m/z* 447.1123 (color code mentioned above). The additional peak pairs for (**2**) represent the CO_2_-depletion and the Na-adduct of rhizobactin B. **c** The corresponding EIC and mass spectra reveal the free ligands of both compounds under Fe-limited conditions

To identify Fe-chelating compounds, the solid phase extract of the supernatant of *Pseudomonas* sp. FEN grown under Fe-limited conditions was screened using HR-MS and DeltaMS (Fig. 2a, b). Fe-pyoverdine-complexes were detected neither in the exponential nor the stationary growth phase under Fe-limited conditions. Due to the structural variety of pyoverdines, we applied an all-ion-fragmentation to identify the characteristic fragments at *m/z* 204 and 230 for potential pyoverdines (Budzikiewicz et al. 2007). Using UHPLC-ESI–MS/MS, none of these fragments was detected in the extract of the supernatant of *Pseudomonas* sp. FEN culture. Moreover, no fluorescence, typical for pyoverdines, was measured in the filtered supernatant of the bacterial growth medium.

**Fig. 3:**
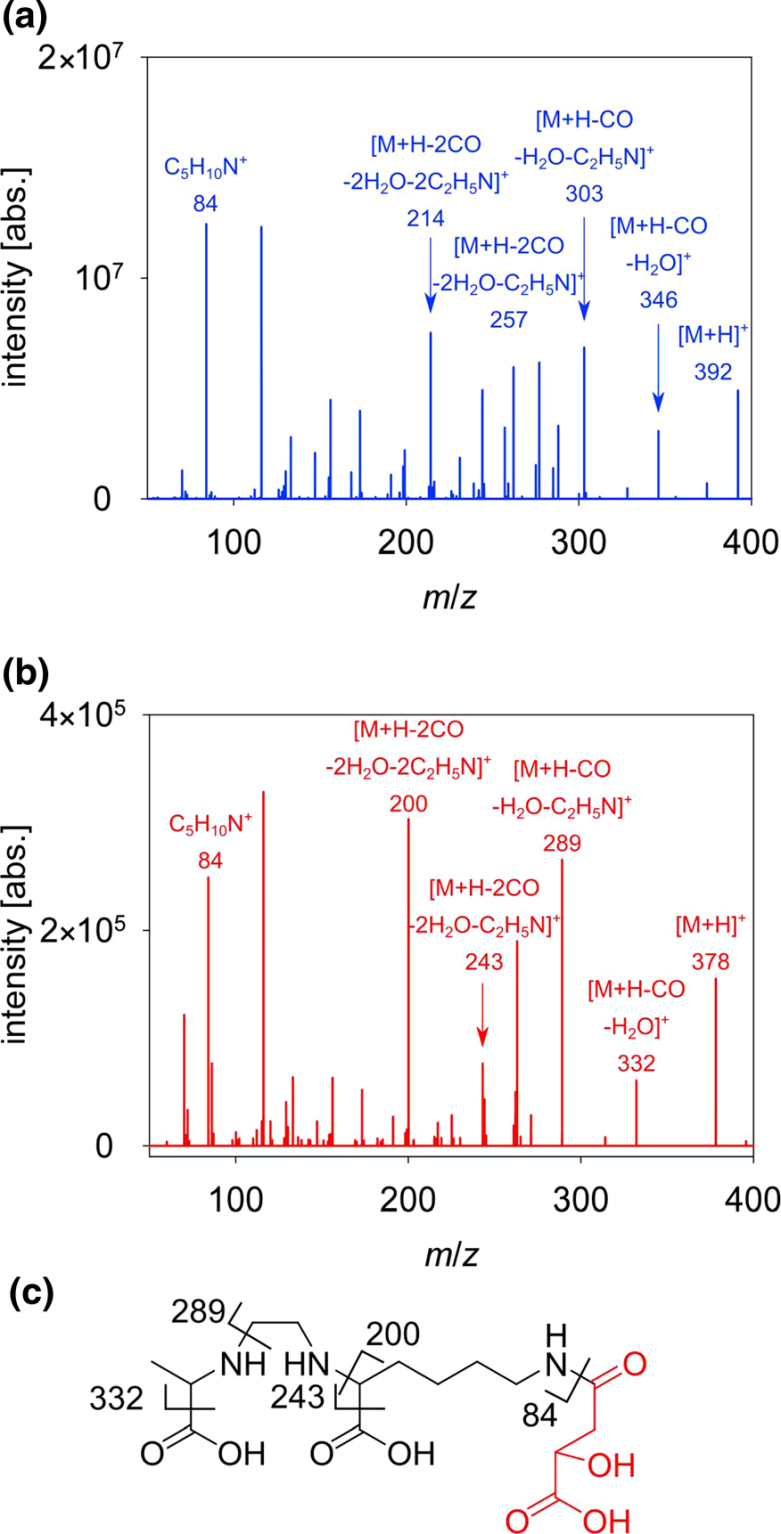
Mass spectrometry (MS/MS) analysis of the identified rhizobactin B (**a**) and rhizobactin (**b**). The structural formula indicates the fragmentation of rhizobactin. **c** The red moiety represents the varied fragment between both compounds. Illustration of fragment assignments of rhizobactin performed by an MS/MS experiment

Due to the absence of pyoverdines, we used MICP to identify Fe-chelating compounds produced by the bacteria. The addition of the Fe-isotopes ^54^Fe and ^58^Fe revealed the presence of two sets of equally intense peaks in the mass spectrum with a mass difference of 3.9937 u, which was detected via DeltaMS in the Fe-limited *Pseudomonas* sp. FEN extract in the stationary phase (Fig. 2a). The HR-MS in ESI + mode indicated Fe-complexing compounds at *m*/*z* of 429.1028 and 433.0964 [M + H]^+^ as well as 443.1186 and 447.1123 [M + H]^+^ for the isotopes ^54^Fe and ^58^Fe, respectively. The other peak pairs represent the CO_2_-depletion and the Na-adduct of the pair at *m*/*z* 443 and 447 (Fig. 2a, b). The free ligands were identified as *m*/*z* 378.1866 [M + H]^+^ (**1**) and 392.2020 [M + H]^+^ (**2**) in the mass spectra without treatment of Fe-isotopes (Fig. 2c). The sum formula for the Fe-free ligand resulted in C_15_H_27_O_8_N_3_ (**1**) and C_16_H_29_O_8_N_3_ (**2**) based on the high-resolution mass, respectively. Both features depicted different but similar compounds due to the small retention time difference. HR-MS indicated that compound (**2**) possesses an additional methyl group in comparison to (**1**). Additional MS/MS experiments confirmed the structural relationship of both compounds showing a similar fragmentation pattern (Fig. 3a, b). The fragmentation pattern revealed the loss of two –COOH and two –C_2_H_5_N until *m*/*z* 200 for (**1**) and 214 for (**2**) for both compounds. Furthermore, there was the loss of one more carboxyl group and a visible C_5_H_10_N-backbone. The assigned sum formula for (**1**) and its fragmentation pattern lead to the assumption it depicted rhizobactin. Due to the similar fragmentation, the additional methyl-group for (**2**) could be localized in the suggested formula (Fig. 3c). Overall, *Pseudomonas* sp. FEN released two ligands complexing Fe, but not pyoverdine or any other siderophore typical for this genus. Siderophores such as enantio-pyochelin, quinolobactin, ornicorrugatin, or pre-pseudomonine were not identified by HR-MS under the applied conditions.

### Structure elucidation of rhizobactin B

**Fig. 4:**
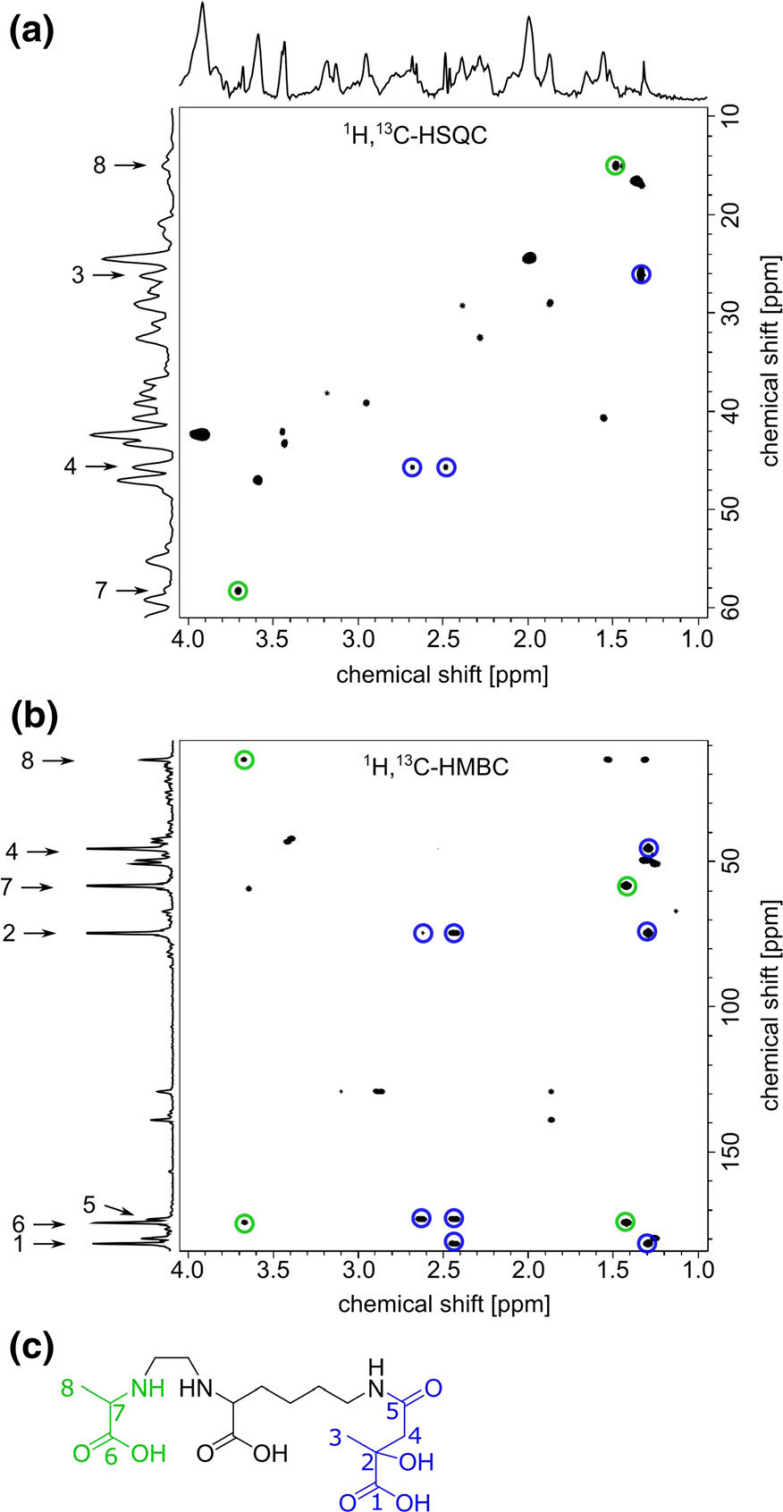
NMR analysis to identify rhizobactin B. **a** ^1^H,^13^C-HSQC (600 MHz), **b** ^1^H,^13^C-HMBC (600 MHz) of the extract in D_2_O and **c** suggested structure of rhizobactin B

To clarify the structural information of the rhizobactin derivative, 2D-NMR experiments were performed with the partly purified compound. In addition to the methyl group of alanine at δ_C_ 14.9 ppm, a second methyl-group was identified at δ_C_ 26.2 ppm (Table 1) which supports the mass spectrometric analysis. The chemical shift of δ_C_ 74.7 ppm and the corresponding ^1^H,^13^C-HSQC with no assigned proton indicated a quaternary carbon atom (C2) coupled to a hydroxyl group (Table 1). The quaternary carbon atoms C1 and C5 were assigned to a carboxyl and an amide group with chemical shifts of δ_C_ 181.6 and 173.1 ppm, respectively. Both carbon atoms C1 and C2 showed direct ^1^H,^13^C-HMBC couplings to H3, and H4, whereas C5 only depicted a coupling to H4. C/H3 represented a methyl group with the typical chemical shift of δ_H_ 1.30 ppm and δ_C_ 26.2 ppm. C/H4 displayed diastereomeric protons of a methylene-bridge at δ_H_ 2.44 and 2.64. Additionally, C3 showed an HMBC coupling with both H4 and C4 with H3. Therefore, we concluded that ^1^H,^13^C-HMBC, and ^1^H,^13^C-HSQC sufficiently revealed the position of the additional methyl-group at the quaternary C2 and belonged to the partial structure of citramalic acid (Fig. 4, Table 1).

**Table 1:** Nuclear magnetic resonance data of rhizobactin B and the annotation of citramalic acid and alanine in D_2_O

C/H	#H	δ_H_ (ppm)	δ_C_ (ppm)	^1^H^13^C-HMBC	Annotation
1	C_q_		181.6	3, 4	Citramalic acid
2	C_q_		74.7	3, 4	Citramalic acid
3	CH_3_	1.30	26.2	4	Citramalic acid
4	CH_2_	2.44 + 2.64	45.7	3	Citramalic acid
5	C_q_		173.1	4	Citramalic acid
6	C_q_		174.4	7, 8	Alanine
7	CH	3.68	58.4	8	Alanine
8	CH_3_	1.44	14.9	7	Alanine

### Growth-dependent production of rhizobactin B and its function as a metallophore

**Fig. 5:**
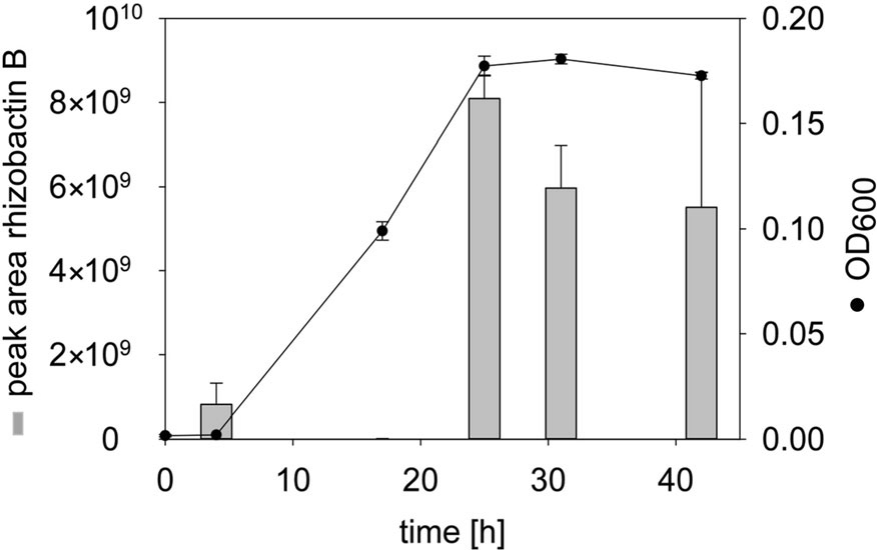
Growth of *Pseudomonas* sp. FEN under Fe-limited conditions. The growth of *Pseudomonas* sp. FEN over time was measured via optical density (OD_600_). The growth-dependent relative content of rhizobactin B (grey bar, in arbitrary units) was measured by UHPLC-HR-MS in the supernatant of the bacterial growth medium after solid-phase extraction at different time points. Error bars represent standard deviations of triplicate measurements

The relative content of rhizobactin B in the supernatant peaked early in the lag phase (t-test, p < 0.05, n = 3) before it ceased when the exponential bacterial growth started (Fig. 5). Only traces of the Fe-complex were detected, due to the low Fe-concentration in the medium and the potential fast uptake by the bacterium. As production and release of rhizobactin B continued through the stationary phase, the highest rhizobactin B content was observed at the beginning of the stationary growth phase (*t*-test, p < 0.001, n = 3). The amount of rhizobactin B released during the remaining 17 h did not change significantly (*t*-test, p > 0.05, n = 3) (Fig. 5).

**Fig. 6:**
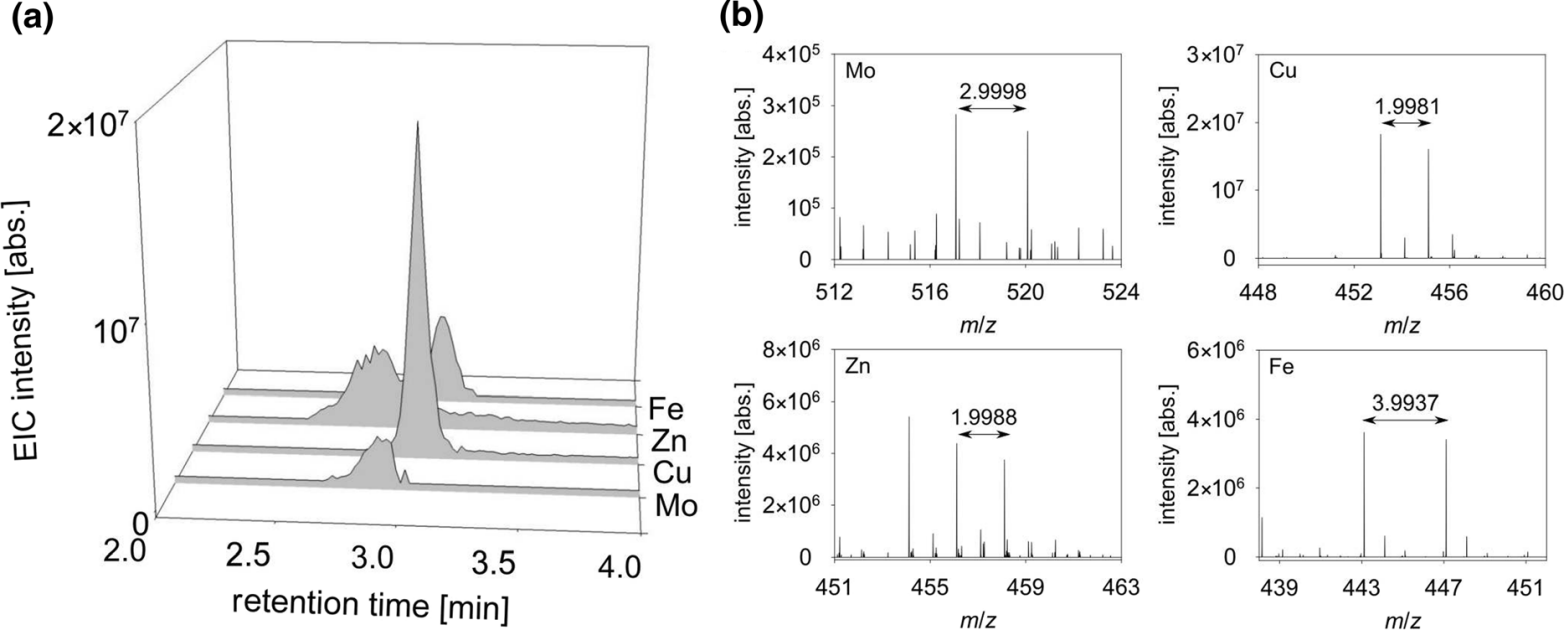
Metal complexation of rhizobactin B. **a** Extracted ion chromatograms (EIC) were created by plotting the intensity of the signal observed at the chosen *m/z* value of metal complexes. They show in vitro complexation of rhizobactin B after the addition of the stable pairs of isotopes ^54^Fe/^58^Fe, ^63^Cu/^65^Cu, ^66^Zn/^68^Zn and ^95^Mo/^98^Mo in a 1:1 ratio. **b** Corresponding mass spectra reveal the according mass shifts

Further DeltaMS analysis revealed the complexation of other metals upon the addition of the isotopes ^63^Cu/^65^Cu, ^66^Zn/^68^Zn, and ^95^Mo/^98^Mo to the SPE of the *Pseudomonas* sp. FEN supernatant (Fig. 6a). In addition to Fe-complexation, mass spectra showed *m*/*z* pairs at 453.1161 and 455.1141 for Cu, 456.1135, and 458.1122 for Zn as well as 517.0823 and 520.0822 for Mo [M + H]^+^ (Fig. 6b). The assigned formulas were C_16_H_27_O_8_N_3_Cu, C_16_H_27_O_8_N_3_Zn, and C_16_H_27_O_10_N_3_Mo, which all belonged to the same ligand, rhizobactin B.

### Rhizobactin B acquires Fe from PWE and the Fe-complex is taken up by the bacterium

**Fig. 7:**
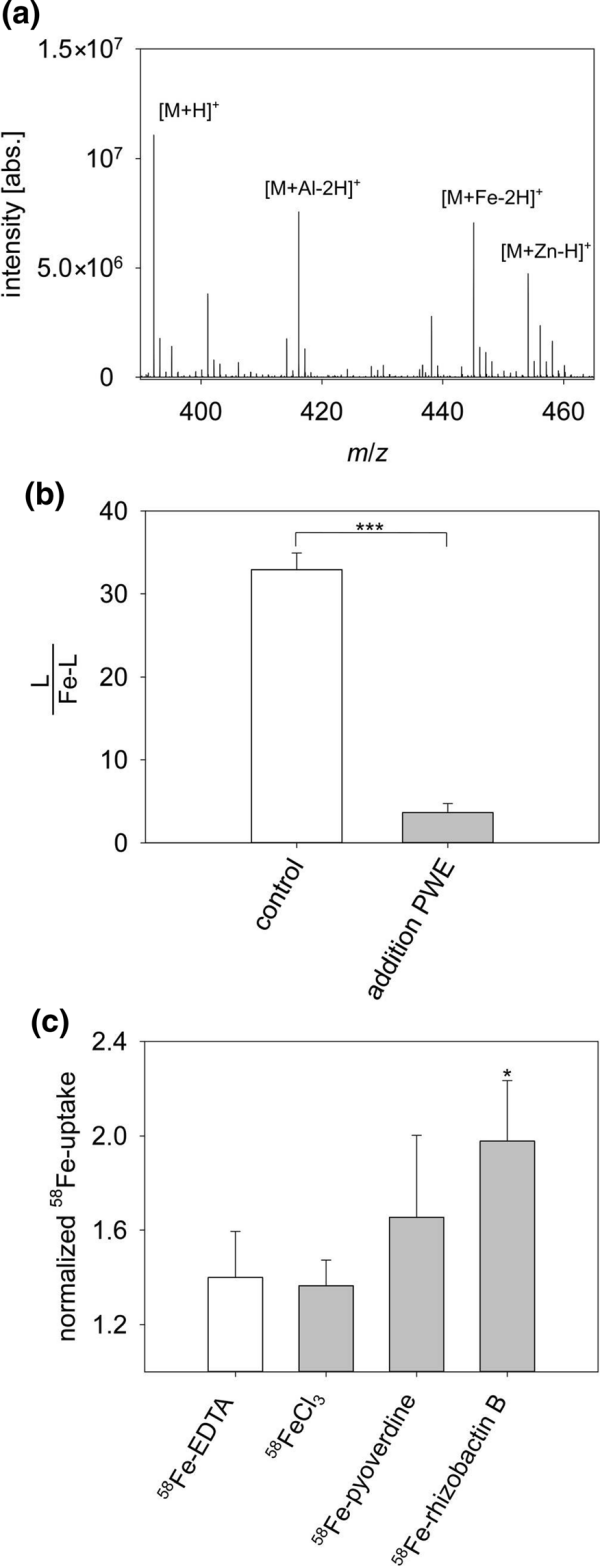
Ligand exchange between rhiozobactin B and Fe-DOM. **a** Mass spectrometric analysis of the peat water extract (PWE) revealed metal complexes of rhizobactin B with Al, Fe, and Zn in the dissolved organic matter (DOM). **b** The decreasing ratio of free ligand to Fe-complex after the addition of PWE containing Fe-DOM confirms the ligand exchange of Fe (t-test, *** p < 0.001, n = 3). Error bars represent standard deviations of three measurements. **c** Uptake experiment of several ^58^Fe-sources (1.0 × 10^–6^ mol L^−1^) in *Pseudomonas* sp. FEN indicated increased Fe-uptake using rhizobactin B (Dunnett’s test in comparison with the control (white bar), p < 0.05, n = 3). Error bars represent standard deviations of triplicate measurements

Furthermore, we tested whether rhizobactin B can acquire Fe and other metals from the PWE obtained from the Fe-rich Schlöppnerbrunnen fen. In addition to Fe, the PWE contained high amounts of Al and Zn, whereas Cu and Mo were negligible or even below the limit of detection (Table 2). After mixing the rhizobactin B with PWE, Al- and Zn-complexes could be identified in addition to Fe-rhizobactin B (Fig. 7a). As DOM entirely complexes Fe in PWE (Kügler et al. 2019), the change of the ratio between ligand and Fe-complex of rhizobactin B could be determined upon the addition of PWE. The stock solution of rhizobactin contained a 30-fold excess of the free ligand to the Fe-complex. After mixing PWE and rhizobactin B, the ratio changes in favor of the Fe-complex, and the amount of free ligand decreases to approximately 10% of the starting value (Fig. 7b).

**Table 2:** Metal concentrations in peat water extract (PWE)

Metals	c (µmol L^−1^)
Al	45.0
Cu	0.03
Fe	18.9
Mo	< LOD
Zn	0.22

Lastly, the physiological function of rhizobactin B as a siderophore for Fe-uptake was confirmed by short-term ^58^Fe uptake experiments with resuspended *Pseudomonas* sp. FEN cell pellets. Indeed, rhizobactin B doubled the Fe-uptake within the first 2 min of incubation (Dunnett’s test, *p < 0.05, n = 3) compared to the uptake of ^58^Fe-EDTA (control experiment), which was not significantly different from 1.0, which means no uptake (*t*-test, p > 0.05, n = 3) (Fig. 7c). Furthermore, we observed that the uptake of ^58^Fe-pyoverdine increased to 1.66 after 2 min, but it was not statistically significant due to the high variation of the uptake measurements.

### *Pseudomonas* sp. FEN genome analysis

Using a draft genome assembly of *Pseudomonas* sp. FEN, we reviewed our chemical analysis and uptake experiments. Importantly, putative genes encoding for the biosynthesis of siderophores, Fe-transport systems, as well as Fe-siderophore sensor proteins and siderophore receptor proteins were found in the *Pseudomonas* sp. FEN genome. For example, genes are present in this genome encoding complete ferrichrome (a hydroxamate siderophore) uptake systems. Additionally, genes encoding various ‘siderophore uptake systems’ were annotated, indicating these systems play a role in the uptake of different siderophores from the surrounding environments, including pyoverdines. Interestingly, two genes encoding homologs to *rhsAB/rhbAB* were annotated as diaminobutyrate-2-oxoglutarate aminotransferase and diaminopimelate decarboxylase in the *Pseudomonas* sp. FEN genome. The *rhsAB/rhbAB* genes are components of the siderophore rhizobactin 1021 biosynthesis gene cluster (*rhbABCDEF/rhsABCDEF*) detected in *Ensifer meliloti* 2011 (formerly (*Sino)Rhizobium meliloti*) (Lynch et al. 2001). The presence of a gene homologous to those encoding the rhizobactin 1021 gene cluster indicates that this particular biosynthetic gene cluster is capable of producing unique siderophores structures that differ in both size and structure, including rhizobactin 1021 and rhizobactin B, in diverse microorganisms, such as *E. meliloti* 2011 and *Pseudomonas* sp. FEN.

Genes encoding ECF sigma factors and surface signaling systems, heme oxygenases, TonB-dependent hemin, ferrichrome receptors, and the *piuCAB* gene cluster, which encodes a Fe-uptake factor and a hydroxamate-type ferrisiderophore receptor, were also found in the *Pseudomonas* sp. FEN genome. Additionally, the genome encodes genes homologous to *pitABC*, a ferric Fe ABC transport system with Fe-binding, ATP-binding, and permease components, and EfeUOB, a ferrous Fe-transport system with a permease, a periplasmic protein along with a peroxidase component. Additionally, genes encoding TonB-dependent receptors in the *Pseudomonas* sp. FEN genome were identified by a manual search of the RAST annotated genome. Nine TonB-dependent receptor genes were identified, along with the closest homologs (Table 3). The homolog with the highest percent identity at the amino acid level was selected for comparison. In addition to TonB-dependent receptors, the *Pseudomonas* sp. FEN genome was examined for genes encoding ECF sigma factors located near the TonB-dependent receptors and genes encoding siderophore-related, iron-related, and heme-related protein families. In total, 4 ECF sigma factor-related genes and the closest homologs were identified (Table S1). The *Pseudomonas* sp. FEN genome contains genes for a variety of siderophore receptors, including ferrichrome receptors, ferrienterochelin receptors, colicin receptors, hemin receptors, and non-specific iron siderophore sensor and receptor proteins, as well as genes encoding both ferric and ferrous iron ABC-type transport systems (Tables 3, S1). Also, the genome contains genes encoding a pyoverdine ABC transporter system, suggesting this strain can utilize exogenous pyoverdine (RAST #2206; Table S1, Fig. 7b). Taken together, the draft genome of *Pseudomonas* sp. FEN provides additional corroborating evidence that this microorganism is capable of active siderophore production, import and export, as well as detection of exogenous siderophores.

**Table 3:** Nine TonB-dependent receptor genes found in the *Pseudomonas* sp. FEN genome with 700 to 900 residues in length and their closest homologs

RAST #	Residues	BLAST hit (Accesion #)	*Pseudomonas*	ID (%)	TonB-dependent functions (homolog)	
183	729	RLJ53657.1	*asplenii*	94	Iron complex outermembrane receptor protein; catecholate-siderophore receptor	CirA
690	736	WP_100941771.1	sp. QS1027	93	TonB-dependent iron receptor	CirA
944	707	WP_177124899.1	*gingeri*	94	TonB-dependent copper receptor	
1454	756	WP_040071009.1	*batumici*	93	TonB-dependent siderophore receptor	Fiu
2180	705	WP_150615638.1	*fluorescens*	93	TonB-dependent siderophore receptor	
2796	861	WP_100944418.1	sp. QS1027	90	TonB-dependent receptor, outer membrane receptor protein, Fe transport	CirA
3107	811	WP_040067439.1	*batumici*	90	TonB-dependent siderophore receptor	
3616	711	WP_152740345.1	sp. MWU12-2323	94	TonB-dependent receptor	
3640	816	WP_100939323.1	sp. QS1027	94	TonB-dependent receptor; ligand-binding site	

## Discussion

Rhizobactin was originally found in *Ensifer meliloti* DM4 (former *Rhizobium meliloti*), a nitrogen-fixing bacterium associated with legumes (Smith and Neilands 1984; Smith et al. 1985). It is structurally different to rhizobactin 1021, a hydroxamate siderophore produced under Fe-stress by *Ensifer meliloti* 2011 (Lynch et al. 2001). The biosynthetic parts of rhizobactin were described as an ethylenediamine group coupled with alanine, lysine, and malic acid (Fig. 3c) (Smith et al. 1985). We thus suggest that the malic acid in rhizobactin is replaced by citramalic acid for the biosynthesis of rhizobactin B, explaining the additional methyl group at C2 (Fig. 4, Table 1). Citramalic acid is known as a bacterial metabolite and building block (Khorassani et al. 2011). Indeed, *P. fluorescens* produces citramalic acid from itaconic acid (Cooper and Kornberg 1962; Nagai 1963).

In general, rhizobactin B belongs to a group of amino polycarboxylic acids, which are known to function as siderophores in bacteria or fungi (Drechsel et al. 1991; Meiwes et al. 1990). Until now, there was only an intermittently described connection between the production of amino polycarboxylic acids acting as siderophore and pseudomonads. Meyer and Hohnadel (1992) reported an increasing growth of several *Pseudomonas* strains in the presence of the xenobiotic nitrolotriacetic acid (NTA) due to the promotion of the Fe-uptake instead of utilizing NTA as carbon or nitrogen source. Interestingly, other similar amino polycarboxylic acids, such as ethylenediaminetetraacetic acid, inhibited *Pseudomonas* sp. growth (Meyer and Hohnadel 1992). Furthermore, genome analysis in recent studies of *P. corrugata* strain RM1-1-4 suggested genes involved in the production of rhizobactin-like siderophores (Zachow et al. 2017). The few overlaps between amino polycarboxylic acids and pseudomonads, as well as the missing pyoverdine production, illustrate the unique profile in the siderophore formation obtained for the *Pseudomonas* strain isolated from the fen. Furthermore, the presence of genes homologous to the rhizobactin 1021 biosynthetic gene cluster in the genome of *Pseudomonas* sp. FEN, as well as the clearly different structure of rhizobactin B compared to rhizobactin 1021, suggests that this biosynthetic gene cluster is used to produce unique siderophores by different microorganisms. However, the mechanisms regulating the production of the unique siderophore compounds produced by this gene cluster during rhizobactin biosynthesis is not known. Further studies will elucidate the biosynthesis of rhizobactin B by deeper analysis of the genome sequence and molecular biological approaches.

Interestingly, *Pseudomonas* sp. FEN began to release rhizobactin B in the early lag phase (Fig. 5), during which Fe was required for growth (Bellenger et al. 2011). In the exponential growth phase, the depletion of rhizobactin B in the growth medium indicates that the Fe-recruitment was faster than its secretion, which underlined the monitored Fe-uptake using rhizobactin B (Fig. 7c). Subsequently, we observed a substantial increase of rhizobactin B in the supernatant during the stationary phase. The reduced demand at the end of bacterial growth might explain the accumulation of the siderophore in the supernatant of the growth medium (Deicke et al. 2013).

Our results show that *Pseudomonas* sp. FEN can acquire Fe via the uptake of Fe-pyoverdine in addition to Fe-rhizobactin B (Fig. 7c). However, pyoverdines were not produced by *Pseudomonas* sp. FEN. The large structural variety of pyoverdines, especially in the amino acid chain structure or length, plays an essential role in the ability of microorganisms to utilize these primary siderophores, whether endogenously or exogenously produced. For instance, up to now, more than 50 pyoverdines of pseudomonads have been identified (Meyer et al. 2008). Strains of *Pseudomonas* were classified into (i) exclusive pyoverdine producers (Matthijs et al. 2009), (ii) pyoverdine producers that also form a secondary siderophore (Youard et al. 2007), (iii) exclusive secondary siderophore producers (Lewis et al. 2004) and (iv) strains without siderophore production (Champomier-Vergès et al. 1996). The inability to produce pyoverdines explains to why *Pseudomonas* sp. FEN does not cluster solely with the other *P. fluorescens* strains in the ANI-based clustering tree (Fig. 1b). Further studies are needed to explore the reasons for the lack of pyoverdine production in *Pseudomonas* sp. FEN, *P. corrugata* strain RM1-1-4 and *P. syringae pv. tomato* strain DC 3200. Up to now, only two different types of non-producers were described: those with a truncated pyoverdine locus and those with an intact, but silent locus (Butaitė et al. 2017). In addition, Hutchins et al. (1991) argued that the N-cost of siderophore production can be too high, therefore, the use of low molecular weight siderophores like rhizobactin B or even pyoverdines from an exogenous source might reflect a strategy to reduce metabolic costs for the biosynthesis of siderophores and Fe-uptake (Cornelis 2010; Matthijs et al. 2009). Siderophores are most useful as an Fe-recycling mechanism in more diffusion-controlled environments, where gradients of siderophore concentration enable its acquisition by a variety of coexisting microorganisms (Hutchins et al. 1991). In this context, Butaité et al. (2017) demonstrated that approximately 9% of the total pseudomonad communities in different soil and aquatic pond environments were not able to produce pyoverdines, however, these non-producers are capable of exploiting pyoverdines released by another organisms to acquire Fe (Butaite et al. 2017). Moreover, the non-pyoverdine producers occurred more frequently in the soil environments (Butaitė et al. 2018). As higher cell densities characterize soil environments compared to aquatic environments, the probability increases significantly of being surrounded by other siderophore producers (Ross-Gillespie et al. 2009; Scholz and Greenberg 2015). While the uptake of pyoverdine is reserved for pseudomonads only (Matthijs et al. 2009), they are flexible in their uptake of siderophores from other resources (Galet et al. 2015). Multiple Fe-uptake systems were identified in the genome of *Pseudomonas* sp. FEN (Table S1). Homologs to the TonB dependent receptor genes identified in the *Pseudomonas* sp. FEN genome which mediate the transport of siderophores were identified in *P. asplenii*, *P. batumici* or *P. fluorescens* (Table 3). TonB-dependent receptors mediate substrate-specific transport across the outer membrane, utilizing energy derived from the inner membrane complex TonB − ExbB − ExbD (Shultis et al. 2006), all of which were found in the *Pseudomonas* sp. FEN genome (Table 3). Several potential TonB systems might be involved in the uptake of siderophores by *Pseudomonas* sp. FEN and must be investigated further. Interestingly, we found some potential encoding TonB-dependent receptor sequences, which were too short to represent active receptors (data not shown). This reduction might be due to a selective pressure from the environment. A similar observation has been made in the case of *P. aeruginosa* isolated from cystic fibrosis lungs (Dingemans et al. 2014).

Previous studies suggest that pseudomonads capable of using various siderophores are more effective scavengers for Fe than those who rely solely on endogenous siderophores (Matthijs et al. 2009). Likewise, a study by Goldberg (2000) argued that the variability in the Fe-uptake and the ability to decrease the metabolic costs depict the high adaptability of pseudomonads, ultimately resulting in the colonization of diverse ecological niches. As Fe-DOM complexes provide the majority of soluble Fe in the Schlöppnerbrunnen fen (Kügler et al. 2019), a ligand exchange must occur to acquire Fe and to overcome Fe-limitations. For example, in the presence of tannic acids, *A. vinelandii* produced a higher amount of metallophores to gain V and Mo to sustain nitrogen fixation (Jouogo Noumsi et al. 2016). In addition to the presence of soluble Fe, PWE, which was filtered through 0.3 µm glass fiber filters, often contains colloidal Fe-forms. These colloids are also known to be associated with DOM (Neubauer et al. 2013; Stolpe et al. 2013). Overall, ligand-associated Fe-acquisition is necessary to overcome the obstacles faces by the microorganisms.

The formation constant of stable Fe-DOM reaches up to 10^14^ L mol^−1^ (Rue and Bruland 1995), whereas Fe-siderophores can form more stable complexes up to 10^52^ L mol^−1^, depending on the ligand (Kraemer 2004; Lalonde et al. 2012). Even though the smaller, secondary siderophores have a lower affinity for Fe (Matthijs et al. 2009), their formation constants are still larger than those of Fe-DOM. The relatively weak formation constant of the hard Fe^III^ Lewis acid with rhizobactin is 10^19^ L mol^−1^ defined by the coordination to the comparatively soft 1,2-diaminoethane side and to the hard oxygen atoms of the carboxyl and hydroxyl groups (Schwyn and Neilands 1987a). These groups of rhizobactin can also coordinate to the borderline Fe^II^ acid (formation constant 10^9^ L mol^−1^) according to the HSAB (hard and soft acids and bases) principle (Neilands 1993; Schwyn and Neilands 1987a). Here, we assume comparable binding properties for rhizobactin B. Indeed, rhizobactin B mixed with PWE outcompeted the DOM and chelated Fe for instant uptake (Fig. 7). Interestingly, although *Pseudomonas mendocina* could utilize Fe-DOM complexes even without the use of siderophores, they increase the availability of accessible Fe-species (Kuhn et al. 2012). We thus conclude that Fe-DOM complexes are readily available for the acquisition of Fe by siderophores as also previously reported (Kuhn and Maurice 2014).

As the metal complexation of rhizobactin B goes beyond Fe-complexation (Fig. 6), and elevated amounts of other metals were detected in the PWE of the Schlöppnerbrunnen fen (Table 2), it is tempting to assume that rhizobactin B also acquires, e.g., Zn from the metal-DOM pool under natural conditions or it is involved in detoxification processes. Nevertheless, the complexation of Fe^III^ seems to be primary as the formation constants of typical metallophores, e.g., for Zn^II^, Cu^II^ and Mn^II^-pyoverdine-complexes are much lower (10^17^ and 10^22^ L mol^−1^) compared to the constant of 10^32^ L mol^−1^ for the Fe^III^-pyoverdine-complex (Chen et al. 1994). Here, we assume similar changes in affinity for the metal complexation of rhizobactin B. The complexation of metals other than Fe, such as Cu, Mo, or Zn, is also in agreement with the mode of action of pyochelin, a secondary siderophore produced by *P. aeruginosa* (Braud et al. 2009; Cunrath et al. 2016), and recently identified metallophores in *Frankia* spp. (Deicke et al. 2019). In this context, we speculate that rhizobactin B and other metallophores can contribute to the metal homeostasis of the most dominating plants, e.g., the *Molinia* grasses, *Sphagnum* mosses and *Carex* sedges, found in the Schlöppnerbrunnen fen (Paul et al. 2006). For example, the phytosiderophore mugineic acid, which is released by graminaceous plants (Sugiura and Nomoto 1984) and structurally related to rhizobactin B, binds Fe^III^, Ni^II^, Cu^II^, or Zn^II^. This zincophore can be useful in recruiting Zn from the soil for Zn-deficient plants (Suzuki et al. 2006). Considering the ligandosphere, which describes the entirety of excreted metal complexing agents (Deicke et al. 2019), future studies will shed light on the interactions between sedges, bacteria and metal-DOM, which might be mediated by metal acquisition from the DOM in the fen.

### Conclusion

The structural identification of siderophores still plays an essential role in the discovery of new natural products, which may be utilized for various medical or environmental applications. *P. fluorescens* strains are well-studied siderophore producers, which are known to produce fluorescent pyoverdines. However, the *Pseudomonas* sp. FEN, which was previously isolated from the Fe-rich Schlöppnerbrunnen fen, shows an unusual pattern in its siderophore production. This strain does not produce any pyoverdine under the studied conditions. *Pseudomonas* sp. FEN secreted a previously unknown (for this genus) Fe-chelating compound, the amino polycarboxylic acid rhizobactin B, utilized to overcome the Fe-limitations which microorganisms often face in nature. The Fe-uptake pattern of *Pseudomonas* sp. FEN using both rhizobactin B and pyoverdine indicates that this bacterium not only utilizes various mechanisms of Fe-acquisition, it also produces a unique variety of siderophores. These characteristics have the potential to enhance the Fe-accessibility and competitiveness of *Pseudomonas* sp. FEN against other microorganisms. The chelation of other metals (e.g., Zn) widens the ecological role of rhizobactin B for *Pseudomonas* sp. FEN*,* which will be investigated further in future studies.

## Electronic supplementary material

Below is the link to the electronic supplementary material.

Supplementary material 1 (PDF 206 kb)

## Abbreviations

ANI: Average nucleotide identity
AAS: Atomic absorption spectroscopy
CAS: Chrome azurol S
DOM: Dissolved organic matter
EDTA: Ethylenediaminetetraacetic acid
ESI: Electrospray ionization
HR-MS: High-resolution mass spectrometry
ICP-MS: Inductively coupled plasma mass spectrometry
MWMM: Modified Wolfe’s mineral medium
NMR: Nuclear magnetic resonance spectroscopy
MICP: Metal isotope-coded profiling
MS: Mass spectrometry
NTA: Nitrolotriacetic acid
PWE: Peat water extract
OD: Optical density
SPE: Solid phase extraction
UHPLC: Ultra high-performance liquid chromatography
