# Supplementary material for: Rhizobactin B is the preferred siderophore by a novel Pseudomonas isolate to obtain iron from dissolved organic matter in peatlands
Source: Biometals. 2020 Oct 7;33(6):415–33. doi: 10.1007/s10534-020-00258-w (PMC7676072; doi:10.1007/s10534-020-00258-w)
Supplement: Supplementary file 1 — Supplementary material 1 (PDF 206 kb) [file 10534_2020_258_MOESM1_ESM.pdf]

Supporting Information for

Rhizobactin B is the preferred siderophore by a novel *Pseudomonas* isolate to obtain iron from dissolved organic matter in peatlands

Stefan Kügler, Rebecca E. Cooper, Johanna Boessneck, Kirsten Küsel, and Thomas Wichard*

Stefan Kügler, Johanna Boessneck and Thomas Wichard*, Institute for Inorganic and Analytical Chemistry (IAAC), Friedrich Schiller University Jena, 07743 Jena, Germany.

Stefan Kügler, Rebecca E. Cooper, Johanna Boessneck and Kirsten Küsel, Institute of Biodiversity, Friedrich Schiller University Jena, 07743 Jena, Germany.

Kirsten Küsel, The German Centre for Integrative Biodiversity Research (iDiv), Halle-Jena-Leipzig, 04103 Leipzig, Germany.

*Corresponding author: Thomas.Wichard@uni-jena.de; Tel.: +49 3641 948184

Thomas Wichard: ORCID 0000-0003-0061-4160

Content

Table S1: *Pseudomonas* sp. FEN FEN genome was searched for TonB-dependent receptors, siderophore-related, iron-related, and heme-related protein families as well as genes encoding ECF sigma factors.

Table S1 *Pseudomonas* sp. FEN genome was searched for TonB-dependent receptors, siderophore-related, iron-related, and heme-related protein families, as well as genes, encoding ECF sigma factors (> 150 residues)

**RAST n#**	**Residues n#**	**BLAST hit (Accesion #)**	***Pseudomonas***	**Id (%)**	**Function (homolog)**	**Abb.**	**ECF**	**TonB**
43	253	WP_052451033.1	*batumici*	97	Uroporphyrinogen-III C-methyltransferase	CobA		
98	143	WP_159897198.1	sp. LD120	86	Biopolymer transporter	ExbD		
99	212	MPQ66032.1	sp. MWU12-2323	97	MotA/TolQ/ExbB proton channel family protein			
183	729	RLJ53657.1	*asplenii*	94	Iron complex outermembrane receptor protein; catecholate-siderophore receptor	CirA		+
440	244	WP_103498954.1	*gingeri*	90	Putative heme iron utilization protein	HugZ		
690	736	WP_100941771.1	sp. QS1027	93	TonB-dependent iron receptor	CirA		+
778	342	WP_177093228.1	*gingeri*	95	Ferrochelatase	HemH		
944	707	WP_177124899.1	*gingeri*	94	TonB-dependent copper receptor			+
945	461	WP_152741573.1	sp. MWU12-2323	93	PepSY domain-containing protein			
946	263	WP_040063952.1	*batumici*	92	ABC-type cobalamin/Fe3+-siderophores transport system	FepC		
947	317	WP_121136378.1	*asplenii*	93	Metal-binding protein; ABC transporter substrate-binding protein	TroA		
948	337	WP_122482160.1	*viridiflava*	89	Iron ABC transporter permease			
982	209	WP_177101963.1	*gingeri*	96	Protein-methionine-sulfoxide reductase heme-binding subunit	MsrQ		

**RAST n#**	**Residues n#**	**BLAST hit (Accesion #)**	***Pseudomonas***	**Id (%)**	**Function (homolog)**	**Abb.**	**ECF**	**TonB**
983	338	WP_040064185.1	*batumici*	97	Protein-methionine-sulfoxide reductase catalytic subunit	MsrP		
1065	155	WP_121135083.1	*asplenii*	97	Bacterioferritin; ferritin-like diiron-binding domain	Bfr		
1192	140	WP_040071270.1	*batumici*	95	Bacterioferritin, ferritin-like diiron-binding domain	Bfr		
1331	296	WP_177103618.1	*gingeri*	99	Protoheme IX farnesyltransferase	CtaB		
1337	394	WP_040070711.1	*batumici*	94	NO-inducible flavohemoprotein	HmpA		
1454	756	WP_040071009.1	*batumici*	93	TonB-dependent siderophore receptor	Fiu		+
1662	3222	WP_081492743.1	sp. M47T1	93	Adhesin			
1665	3222	WP_152740400.1	sp. MWU12-2323	85	Filamentous hemagglutinin N-terminal domain-containing protein			
1841	212	WP_040065757.1	*batumici*	95	Cytochrome c biogenesis heme-transporting ATPase	CcmA		
1842	223	WP_103499452.1	sp. MWU12-2323	99	Heme exporter protein	CcmB		
1843	252	WP_177094887.1	*gingeri*	96	Cytochrome c biogenesis protein	CcmC/CcsA		
1844	59	WP_040065763.1	*batumici*	100	Heme exporter protein	CcmD		
1845	152	WP_077045806.1	sp. KK4	98	Cytochrome c maturation protein	CcmE		
1846	663	WP_177145195.1	*gingeri*	98	Heme lyase CcmF/NrfE family subunit	CcmF		
1847	179	WP_152737035.1	sp. MWU12-2323	94	DsbE family thiol:disulfide interchange protein	CcmG		
1848	157	WP_040065772.1	*batumici*	93	Cytochrome c-type biogenesis protein CcmH	CcmL/CcmH		

RAST n#	Residues n#	BLAST hit (Accesion #)	*Pseudomonas*	Id (%)	Function (homolog)	Abb.	ECF	TonB
1849	400	WP_177060461.1	*gingeri*	91	c-type cytochrome biogenesis protein CcmI	CcmH/CcmI		
1918	72	WP_177125739.1	*gingeri*	93	cbb3-type cytochrome oxidase assembly protein	CcoS		
1919	817	WP_177145161.1	*gingeri*	96	Heavy metal translocating P-type ATPase metal-binding domain-containing protein			
1920	180	WP_177095016.1	*gingeri*	94	FixH family protein	FixH		
1921	472	WP_177140487.1	*gingeri*	95	Cytochrome c oxidase accessory protein	CcoG		
1922	325	WP_103032486.1	*gingeri*	96	Cytochrome-c oxidase, cbb3-type subunit III	CcoP		
1923	62	WP_065887892.1	sp. 31 R 17	93	CcoQ/FixQ family Cbb3-type cytochrome c oxidase assembly chaperone	CcoQ/FixQ		
1924	203	WP_025110367.1	sp. H1h	99	Cytochrome-c oxidase, cbb3-type subunit II	CcoO		
1925	481	WP_152737003.1	sp. MWU12-2323	99	Cytochrome-c oxidase, cbb3-type subunit I	CcoN		
1926	313	RBH55338.1	sp. MWU12-2324	94	Cytochrome-c oxidase, cbb3-type subunit III	CcoP		
1927	66	WP_040064645.1	*batumici*	91	Cytochrome cbb-3 oxidase	CcoQ		
1928	203	WP_177095029.1	*gingeri*	94	Cytochrome-c oxidase, cbb3-type subunit II	CcoO		
1929	475	WP_177105566.1	*gingeri*	97	Cytochrome-c oxidase, cbb3-type subunit I	CcoN		
2080	465	WP_177105245.1	*gingeri*	97	Uroporphyrinogen-III C-methyltransferase	CobA		
2180	705	WP_150615638.1	*fluorescens*	93	TonB-dependent siderophore receptor			+

RAST n#	Residues n#	BLAST hit (Accesion #)	*Pseudomonas*	Id (%)	Function (homolog)	Abb.	ECF	TonB
2205	302	WP_038414296.1	*cremoricolorata*	90	Metal ABC transporter permease; ABC-type Mn2+/Zn2+ transport system, permease	ZnuB		
2206	227	WP_069076107.1	*fluorescens*	85	Metal ABC transporter ATP-binding protein in pyoverdine cluster			
2336	450	WP_124335016.1	*chlororaphis*	78	Coproporphyrinogen III oxidase, HemN C-terminal domain	HemN_C		
2337	463	WP_077430437.1	sp. C9	88	Oxygen-independent coproporphyrinogen III oxidase	HemN		
2339	280	WP_052964445.1	*syringae*	81	Uroporphyrinogen-III C-methyltransferase	CobA		
2340	393	WP_129444728.1	ACM7	87	Heme d1 biosynthesis radical SAM protein	NirJ		
2341	168	WP_123722304.1	*lini*	89	Lrp/AsnC family transcriptional regulator			
2342	148	WP_150804542.1	*fluorescens*	83	Lrp/AsnC family transcriptional regulator			
2343	173	WP_008075026.1	sp. GM79	77	Lrp/AsnC family transcriptional regulator; DNA-binding transcriptional regulator			
2344	153	KTC33597.1	sp. ABAC61	83	AsnC family transcriptional regulator			
2345	393	WP_111454566.1	sp. URMO17WK12:I6	88	Protein NirF; cytochrome D1 heme domain	NirF		
2794	173	RBH56222.1	sp. MWU13-2860	92	RNA polymerase sigma factor		+	
2795	322	WP_121134120.1	*asplenii*	92	FecR domain-containing protein	FecR		
2796	861	WP_100944418.1	sp. QS1027	90	TonB-dependent receptor; Outer membrane receptor protein, Fe transport	CirA		+

RAST n#	Residues n#	BLAST hit (Accesion #)	*Pseudomonas*	Id (%)	Function (homolog)	Abb.	ECF	TonB
2797	203	WP_177123997.1	*gingeri*	91	Biliverdin-producing heme oxygenase	HemO		
2846	474	WP_040070318.1	*batumici*	92	c-type cytochrome; Diheme oxidoreducatase, putative peroxidase			
2876	73	WP_040070349.1	*batumici*	96	Bacterioferritin-associated ferredoxin	Bfd		
2877	157	RBH56294.1	sp. MWU13-2860	97	Bacterioferritin; Ferritin-ike Diiron-binding domain	Bfr		
2882	350	WP_177123974.1	*gingeri*	91	ATP-binding cassette domain-containing protein; ABC-type Fe3+/spermidine/putrescine transport systems, ATPase component			
3102	617	WP_040067449.1	*batumici*	91	IucA/IucC family siderophore biosynthesis protein			
3105	172	WP_040067443.1	*batumici*	91	sigma-70 family RNA polymerase sigma factor		+	
3106	326	WP_040067441.1	*batumici*	84	FecR family protein; Periplasmic ferric-dicitrate binding protein	FecR		
3107	811	WP_040067439.1	*batumici*	90	TonB-dependent siderophore receptor			+
3108	397	WP_040067438.1	*batumici*	92	ABC transporter substrate-binding protein; ferrichrome/ferrioxamine B periplasmic transporter	TroA superfamily		
3109	354	WP_131063491.1	sp. BGI-2	91	Iron ABC transporter permease; ABC-type Fe3+-siderophore transport system, permease component			
3188	339	WP_017129082.1	*gingeri*	95	Iron ABC transporter substrate-binding protein			
3189	538	WP_177102669.1	*gingeri*	95	ABC transporter permease subunit			

RAST n#	Residues n#	BLAST hit (Accesion #)	*Pseudomonas*	Id (%)	Function (homolog)	Abb.	ECF	TonB
3190	359	WP_121141315.1	*asplenii*	93	ABC transporter ATP-binding protein; ABC-type Fe3+/spermidine/putrescine transport system, ATPase component			
3238	325	WP_103499260.1	*gingeri*	99	Porphobilinogen synthase			
3253	2212	WP_177098858.1	*gingeri*	91	Hemagglutinin repeat-containing protein			
3254	173	WP_121139294.1	*asplenii*	91	Toxin-activating lysine-acyltransferase	HlyC		
3495	553	WP_177143682.1	*gingeri*	98	Nitrite/sulfite reductase			
3600	134	WP_150573043.1	*fluorescens*	88	Biopolymer transporter	ExbD		
3601	242	WP_079303192.1	sp. Ep R1	96	MotA/TolQ/ExbB proton channel family protein	ExbB/TolQ		
3616	711	WP_152740345.1	sp. MWU12-2323	94	TonB-dependent receptor			+
3640	816	WP_100939323.1	sp. QS1027	94	TonB-dependent receptor; ligand-binding site			+
3748	83	WP_177130255.1	*gingeri*	95	Hemagglutinin repeat-containing protein			
3750	4313	WP_177141005.1	*gingeri*	88	Hemagglutinin repeat-containing protein			
3942	142	WP_177101709.1	*gingeri*	98	Biopolymer transporter			
3943	615	WP_177145530.1	*gingeri*	93	DUF2341 domain-containing protein			
3978	281	WP_177145518.1	*gingeri*	95	FTR1 family protein; high affinity ferrous iron permease	FTR1/Fip1/EfeU		
3979	398	WP_040067323.1	*batumici*	90	Iron uptake system protein; ferrous iron transporter periplasmic, cupredoxin-like domain	EfeO		

RAST n#	Residues n#	BLAST hit (Accesion #)	*Pseudomonas*	Id (%)	Function (homolog)	Abb.	ECF	TonB
3980	439	WP_177101670.1	*batumici*	93	Deferrochelatase/peroxidase	EfeB		
3981	275	WP_152738845.1	sp. MWU12-2323	97	Iron uptake system protein; ferrous iron transporter periplasmic, cupredoxin-like domain	EfeO		
4019	317	WP_100941090.1	sp. QS1027	90	DUF4880 domain-containing protein	FecR		
4020	183	WP_177098074.1	*gingeri*	98	sigma-70 family RNA polymerase sigma factor		+	
4067	568	WP_040069813.1	*batumici*	92	Cyclic peptide export ABC transporter			
4088	151	WP_040070281.1	*batumici*	91	Biliverdin-producing heme oxygenase; heme ligand			
4095	267	WP_121134120.1	*asplenii*	93	FecR domain-containing protein	FecR		
4116	99	WP_121134120.1	*asplenii*	82	FecR domain-containing protein	FecR		
4117	78	WP_084615120.1	*batumici*	93	FecR domain-containing protein	FecR		
4118	81	WP_177143386.1	*gingeri*	96	FecR domain-containing protein	FecR		
4121	63	WP_177143386.1	*gingeri*	97	FecR domain-containing protein	FecR		
4122	240	WP_121134120.1	*asplenii*	89	FecR domain-containing protein	FecR		
4123	173	RBH56222.1	sp. MWU13-2860	92	RNA polymerase sigma factor		+	
4138	177	WP_177103950.1	*gingeri*	89	FecR domain-containing protein	FecR		
3944	543	WP_178129836.1	sp. MWU12-2323	96	ShlB/FhaC/HecB family hemolysin secretion/activation protein			

RAST n#	Residues n#	BLAST hit (Accesion #)	*Pseudomonas*	Id (%)	Function (homolog)	Abb.	ECF	TonB
4557	1604	WP_152740400.1	sp. MWU12-2323	90	Filamentous hemagglutinin N-terminal domain-containing protein			
4558	274	WP_177141023.1	*gingeri*	74	Hemagglutinin repeat-containing protein			
4638	213	WP_152740400.1	sp. MWU12-2323	62	Filamentous hemagglutinin N-terminal domain-containing protein			
4639	113	WP_177104149.1	*gingeri*	72	Filamentous hemagglutinin N-terminal domain-containing protein			
4680	285	WP_124421089.1	sp. R2-60-08W	66	Iron ABC transporter permease			
4681	103	WP_017130562.1	*agarici*	67	Iron ABC transporter permease; ABC-type Fe3+ transport system, permease component	FbpB		
4683	211	WP_161905978.1	sp. L13	54	ABC transporter permease subunit			
5085	307	WP_040062961.1	*batumici*	97	Oxygen-dependent coproporphyrinogen oxidase; coproporphyrinogen III oxidase	HemF		
5123	249	WP_121135748.1	*asplenii*	94	SURF1 family protein; cytochrome c oxidase (complex IV)			
5125	360	RBH58670.1	sp. MWU13-2860	98	Heme A synthase; Cytochrome oxidase assembly protein	Cox15-CtaA		
5126	300	WP_100939995.1	sp. QS1027	98	Protoheme IX farnesyltransferase	CtaB		
5265	334	WP_152736737.1	sp. MWU12-2323	95	Extracellular solute-binding protein			
5266	536	WP_121135415.1	*asplenii*	96	Iron ABC transporter permease; ABC-type Fe3+ transport system, permease component			

RAST n#	Residues n#	BLAST hit (Accesion #)	***Pseudomonas***	**Id (%)**	**Function (homolog)**	**Abb.**	**ECF**	**TonB**
5321	359	RBH53541.1	sp. MWU13-2860	95	Putative 2-aminoethylphosphonate ABC transporter ATP-binding protein	PhnT		
5322	575	WP_040071539.1	*batumici*	95	Putative 2-aminoethylphosphonate ABC transporter permease subunit	PhnU		
5323	341	WP_121135351.1	*asplenii*	97	Putative 2-aminoethylphosphonate ABC transporter substrate-binding protein	PhnS		
5369	401	WP_121135314.1	*asplenii*	98	Radical SAM family heme chaperone HemW; HemN family oxidoreductase	HemW		
5606	143	WP_121135101.1	*asplenii*	97	Protoporphyrinogen oxidase	HemJ		
5612	209	WP_177101963.1	*gingeri*	96	Protein-methionine-sulfoxide reductase heme-binding subunit	MsrQ		
5684	136	WP_040064097.1	*batumici*	96	Ferric iron uptake transcriptional regulator			
5891	356	WP_040063677.1	*batumici*	99	Uroporphyrinogen decarboxylase	HemE		
6129	338	WP_177094115.1	*gingeri*	99	Porphobilinogen synthase	HemB		
6145	408	WP_040071647.1	*batumici*	95	Heme biosynthesis protein	HemY		
6146	376	WP_177145349.1	*gingeri*	94	Uroporphyrinogen-III C-methyltransferase			
6147	256	WP_177145348.1	*gingeri*	93	Uroporphyrinogen-III synthase			
6148	314	WP_177094092.1	*gingeri*	97	Hydroxymethylbilane synthase	HemC		
